# Disease Knowledge Assessed by RAKE-T and Its Sociodemographic and Clinical Correlates in Patients with Rheumatoid Arthritis Receiving Biologic and Targeted Synthetic DMARDs: A Multicenter Cross-Sectional Study

**DOI:** 10.3390/jcm15208178

**Published:** 2026-10-21

**Authors:** Nihal Lermi, Eyyüp Garip, Abdullah Yusuf İstanbullu, Lalenur Akyüz, Beyza Us Demirelli, Fatih Albayrak, Burcu Yağız, Mehmet Engin Tezcan, Ediz Dalkılıç, Yavuz Pehlivan, Belkıs Nihan Coşkun

**Affiliations:** 1 Department of Rheumatology, Kartal Dr. Lutfi Kirdar City Hospital, University of Health Sciences, Istanbul 34865, Turkey; 2 Department of Internal Medicine, Faculty of Medicine, Kafkas University, Kars 36000, Turkey; 3 Division of Rheumatology, Faculty of Medicine, Uludag University, Bursa 16059, Turkey; 4 Department of Internal Medicine, Bursa Yuksek Ihtisas Training and Research Hospital, University of Health Sciences, Bursa 16310, Turkey; 5 Division of Rheumatology, Faculty of Medicine, Gaziantep University, Gaziantep 27310, Turkey

**Keywords:** rheumatoid arthritis, disease knowledge, RAKE-T, patient education, biologic DMARDs, targeted synthetic DMARDs, health literacy

## Abstract

**Background/Objectives:** Disease-related knowledge is important in rheumatoid arthritis (RA), particularly for patients receiving biologic (bDMARD) or targeted synthetic (tsDMARD) disease-modifying antirheumatic drugs. This study aimed to assess disease knowledge using the Turkish version of the Rheumatoid Arthritis Knowledge Assessment Questionnaire (RAKE-T) in patients receiving bDMARD/tsDMARD therapy and to investigate its sociodemographic and clinical correlates. **Methods:** This multicenter cross-sectional study included 230 patients with RA receiving bDMARD/tsDMARD therapy at four rheumatology centers. Sociodemographic and clinical characteristics were recorded, and disease knowledge was assessed using the 45-item RAKE-T. Linear or robust regression analyses, as appropriate, were used to evaluate factors associated with total and domain-specific scores, except for the single-item comorbidity domain, which was analyzed using binary logistic regression. **Results:** The mean total RAKE-T score was 61.77 ± 19.00. Older age was associated with lower total RAKE-T and disease knowledge domain scores (*p* = 0.033 for both). University education or higher was associated with higher total RAKE-T scores (β = 14.503, *p* < 0.001) and higher scores in the disease knowledge, pharmacological treatments, non-pharmacological treatments, and adaptive skills domains. Place of residence was associated with disease knowledge, self-care, and adaptive skills scores. Disease duration, duration of biologic therapy, sex, marital status, and previous biologic agent use were not significantly associated with total or domain-specific RAKE-T scores. **Conclusions:** Among patients with RA receiving bDMARD/tsDMARD therapy, disease knowledge was associated with age, educational level, and place of residence, whereas no statistically significant associations were observed with disease duration or duration of biologic therapy. These findings suggest that longer disease or treatment experience should not be assumed to correspond to greater disease knowledge and support periodic reassessment of educational needs and individually tailored patient education.

## 1. Introduction

Rheumatoid arthritis (RA) is a chronic, immune-mediated inflammatory joint disease characterized by progressive synovitis and, if inadequately controlled, structural joint damage and functional impairment. Management of RA typically requires long-term pharmacological treatment, regular rheumatology follow-up, and active patient participation in disease monitoring and self-management [1]. Given the chronic and evolving nature of the disease, maintaining an adequate understanding of the disease, its treatment, and potential complications may be particularly important throughout long-term follow-up.

Disease-related knowledge is considered an important component of comprehensive RA care, as it may support patients’ understanding of their treatment, adherence to prescribed regimens, recognition of potential adverse effects, and engagement in self-management strategies. In recognition of this, the European Alliance of Associations for Rheumatology (EULAR) has emphasized patient education as an integral component of the standard of care for individuals with inflammatory arthritis [2]. Adequate disease knowledge may be particularly relevant for patients receiving complex treatment regimens, such as biologic and targeted synthetic disease-modifying antirheumatic drugs (bDMARDs/tsDMARDs), which require specific knowledge regarding administration, safety monitoring, and long-term management.

To systematically evaluate disease-related knowledge in patients with RA, several assessment instruments have been developed. Among these, the Rheumatoid Arthritis Knowledge Assessment Questionnaire (RAKE) was developed as a comprehensive, self-administered instrument covering disease knowledge, pharmacological and non-pharmacological treatments, comorbidity, self-care, and adaptive skills [3]. RAKE has since been culturally adapted and validated in other languages, and the Turkish version of the questionnaire (RAKE-T) was subsequently validated in Turkish patients with RA [4].

Since its development, RAKE and RAKE-T have been used in a limited number of studies to assess disease-related knowledge, including in the context of educational interventions [5]. In addition, disease-related knowledge in RA has been examined using a range of other locally developed or adapted assessment instruments across different populations [6,7,8,9,10]. However, findings regarding the sociodemographic and clinical factors associated with disease knowledge have not been fully consistent across these studies, with some reporting significant associations with factors such as age or educational level and others finding no such associations. Only a limited number of studies have specifically addressed patients receiving biologic or targeted synthetic therapy.

Patients receiving bDMARD/tsDMARD therapy represent a population in which disease-related knowledge may be of particular clinical relevance, given the treatment-specific information required regarding administration, adherence, and safety, as well as the need for regular rheumatology follow-up [11]. However, whether longer disease duration or longer experience with biologic therapy is accompanied by a correspondingly greater level of disease knowledge, or whether educational needs persist or evolve independently of treatment experience, has not been well established. Clarifying the sociodemographic and clinical correlates of disease knowledge in this specific population may help identify patients with greater educational needs and inform the periodic reassessment of these needs during routine follow-up.

The aim of this multicenter study was to assess disease knowledge using RAKE-T among patients with RA receiving bDMARD/tsDMARD therapy and to investigate the sociodemographic and clinical factors associated with disease knowledge.

## 2. Materials and Methods

This multicenter cross-sectional study was conducted in accordance with the principles of the Declaration of Helsinki and was approved by the Kafkas University Faculty of Medicine Non-Interventional Research Ethics Committee (24 June 2025; decision no. 2025/06/22). The study included a total of 230 patients with RA who were followed at the Rheumatology Outpatient Clinics of Kars Harakani State Hospital and University of Health Sciences Kartal Dr. Lütfi Kırdar City Hospital, the Division of Rheumatology at Bursa Uludağ University Faculty of Medicine, and the Division of Rheumatology at Gaziantep University Faculty of Medicine between June 2025 and July 2026.

Patients were included if they were aged ≥18 years, had been diagnosed with RA according to the 2010 American College of Rheumatology/European Alliance of Associations for Rheumatology (ACR/EULAR) classification criteria, were followed at a rheumatology outpatient clinic, were receiving treatment with a bDMARD or tsDMARD, agreed to participate in the study, and completed the questionnaire in full. Patients who declined to participate, did not complete the questionnaire in full, or had cognitive or communication difficulties that could interfere with the assessment were excluded from the study.

Demographic characteristics, disease-related clinical features, and treatment information were recorded by the investigators during face-to-face interviews using a standardized data collection form. The presence of deformity was assessed by physical examination. The data collection form included age, sex, educational level (illiterate, literate, primary school, middle school, high school, university or higher), marital status (married/single), place of residence (village, district, city), having children, number of children, current smoking status, family history of rheumatic disease, disease duration (months), current bDMARD/tsDMARD treatment, total duration of biologic therapy (months), and history of previous biologic agent use.

Sample-size adequacy was evaluated based on the multivariable regression analyses. According to Green’s recommendations, the minimum sample size was calculated using N ≥ 50 + 8k for testing the overall model and N ≥ 104 + k for testing individual regression coefficients, where k represents the number of estimated regression coefficients (excluding the intercept). With eight independent variables, represented by nine regression coefficients (k = 9) because the three-category place of residence variable required two dummy-coded coefficients, the corresponding minimum sample sizes were 122 and 113, respectively. The final sample of 230 participants exceeded both recommended minimum sample sizes [12].

All patients included in the study were receiving bDMARD or tsDMARD therapy. As part of routine rheumatology practice, all patients received standardized education and counseling before the initiation of these treatments regarding treatment administration, treatment adherence, potential adverse effects, and the follow-up process.

Disease knowledge in patients with RA was assessed using RAKE-T. The questionnaire was originally developed by Rodère et al., and its Turkish validity and reliability study was conducted by Pehlivan et al. [3,4]. RAKE-T consists of 45 items across six domains: disease knowledge (10 items), pharmacological treatments (14 items), non-pharmacological treatments (7 items), comorbidity (1 item), self-care (5 items), and adaptive skills (8 items). Each item is answered as “True,” “False,” or “I don’t know”; correct answers are scored as 1 point, whereas incorrect and “I don’t know” responses are scored as 0 points. Negatively worded items are reverse scored. For the present study, the total score was converted to a 0–100 scale by multiplying the number of correct responses by 100/45 (approximately 2.22 points per correct response). Domain scores were calculated by applying the same weighting to the number of correct responses within each domain. Higher scores indicate a higher level of disease knowledge. The RAKE-T questionnaire was administered face-to-face by the researchers at all participating centers. All questions were read aloud to each participant, including those who were illiterate, and participants’ responses were recorded by the researchers. The same administration procedure was followed across all centers, without providing hints or prompting participants toward particular answers. 

Data were analyzed using IBM SPSS Statistics version 23 (IBM Corp., Armonk, NY, USA) and R version 4.5 (R Foundation for Statistical Computing, Vienna, Austria). The normality of data distribution was assessed using the Kolmogorov–Smirnov test, and the normality of the regression residuals was assessed using the Kolmogorov–Smirnov test with Lilliefors correction and the Shapiro–Wilk test. Multivariable linear or robust regression analysis was used to evaluate factors associated with the total RAKE-T and domain scores (except for the comorbidity domain); the choice between these models was based on regression diagnostics, as described below. Because the comorbidity domain consists of a single item and can take only two values (0 or 2.22), it was analyzed as a binary outcome using multivariable binary logistic regression with the same independent variables; results were expressed as odds ratios (ORs) with 95% confidence intervals, and model fit was summarized using the Cox & Snell and Nagelkerke R^2^ values. Continuous variables were presented as mean ± standard deviation or median (minimum–maximum), as appropriate, whereas categorical variables were presented as frequencies and percentages. The independent variables included in the multivariable regression models were selected a priori based on the relevant literature and clinical relevance. Sociodemographic variables (age, sex, education level, marital status, and place of residence) and clinical/treatment-related variables (disease duration, duration of biologic therapy, and previous biologic agent use) were entered simultaneously into the models to account for their potential confounding effects. For the multivariable regression analyses, educational level was dichotomized as high school or lower versus university or higher to distinguish participants with and without higher education and to provide a parsimonious, interpretable comparison. Regression model assumptions were assessed using model diagnostics. Linearity and homoscedasticity were evaluated using residual plots, multicollinearity was assessed using variance inflation factors (VIFs), and influential observations and outliers were examined using Cook’s distance and standardized residuals. Residual distributions were also evaluated. Model selection was based on these regression diagnostics rather than solely on the distribution of the dependent variable: classical linear regression was retained when the residuals did not depart significantly from normality, whereas robust regression was used when the residuals departed significantly from normality, in order to reduce the influence of observations with large residuals on the coefficient estimates. The diagnostic findings for each outcome are reported in the Section 3. Robust regression models were estimated using Huber’s M-estimator (MASS::rlm). Model fit was reported as R^2^, adjusted R^2^, and the overall F test for the linear regression models, and as weighted R^2^ and weighted adjusted R^2^ for the robust regression models; for the robust regression models, these measures were calculated by refitting the models by weighted least squares (WLS) using the final Huber M-estimator weights and are presented for descriptive purposes only. Because this refitting does not provide a valid overall significance test for the original robust regression models, no global F test or model *p* value is reported for the robust models; inference for these models is based on the coefficient-level tests described below. For the robust regression models, Huber’s M-estimator was applied with a tuning constant of c = 1.345 and the median absolute deviation as the scale estimate; 95% confidence intervals and *p* values for the regression coefficients were calculated from the asymptotic standard errors using Wald-type t tests with N − k − 1 degrees of freedom, where k is the number of estimated regression coefficients excluding the intercept (k = 9). In a sensitivity analysis, recruitment center was additionally entered into each model as a categorical covariate, in addition to the existing independent variables (reference category: Kars; other centers: Gaziantep, Kartal, and Uludağ), to assess whether the associations, particularly those related to place of residence, persisted after adjustment for center; for each outcome, the same type of model was used as in the main analysis. The total RAKE-T score was considered the primary outcome, whereas analyses of the individual RAKE-T domains were considered secondary and exploratory. No formal adjustment for multiple testing was applied to the domain-specific analyses because of the interrelated nature of the domains and the potential increase in Type II error. Accordingly, findings from these exploratory analyses were interpreted with caution. A *p* value < 0.05 was considered statistically significant.

## 3. Results

A total of 230 patients with RA were included in the study. Of these, 177 (77.0%) were female. The median age was 57 years (minimum–maximum: 18–77), the median number of children was 2 (minimum–maximum: 0–9), the median disease duration was 168 months (minimum–maximum: 6–516), and the median total duration of biologic therapy was 36 months (minimum–maximum: 3–252). Of the patients, 190 (82.6%) had a high school education or lower, whereas 40 (17.4%) had a university education or higher. A total of 178 (77.4%) patients were married, 163 (70.9%) lived in a city center, and 200 (87.0%) had children. Fifty-five (23.9%) patients were current smokers. Disease duration was ≥5 years in 199 (86.5%) patients, and 102 (44.4%) had a history of previous biologic agent use. A family history of rheumatic disease was present in 100 (43.5%) patients, and deformity was identified in 73 (31.7%) patients (Table 1).

The bDMARD and tsDMARD agents used by the patients are shown in Table 2. The most commonly used treatment was adalimumab (ADA), which was used by 61 (26.5%) patients, followed by rituximab (RTX) in 55 (23.9%), tocilizumab (TCZ) in 32 (13.9%), and etanercept (ETN) in 23 (10.0%) patients.

The mean total RAKE-T score was 61.77 ± 19.00. The median scores for the disease knowledge, pharmacological treatments, non-pharmacological treatments, comorbidity, self-care, and adaptive skills domains were 15.55, 17.77, 8.88, 0, 6.66, and 13.33, respectively (Table 3).

Regression diagnostics are summarized as follows. Variance inflation factors ranged from 1.049 to 2.462 in the linear regression models and from 1.046 to 2.498 in the robust regression models, all well below the commonly used threshold of 10, with the highest values observed for the two place of residence dummy variables. The residuals did not depart significantly from normality for the total RAKE-T score (Kolmogorov–Smirnov *p* = 0.091; Shapiro–Wilk *p* = 0.592) or for the disease knowledge (*p* ≥ 0.200; *p* = 0.435), pharmacological treatments (*p* ≥ 0.200; *p* = 0.500), and non-pharmacological treatments (*p* = 0.086; *p* = 0.141) domains; these outcomes were therefore analyzed using linear regression. In contrast, the residuals departed significantly from normality for the self-care (*p* = 0.002; *p* < 0.001) and adaptive skills (*p* < 0.001; *p* < 0.001) domains, which were therefore analyzed using robust regression. The maximum Cook’s distance values were 0.046 for the total RAKE-T score and 0.042, 0.037, 0.032, 0.040, and 0.051 for the disease knowledge, pharmacological treatments, non-pharmacological treatments, self-care, and adaptive skills domains, respectively; no observation approached a Cook’s distance of 1, and the maximum values were of similar magnitude in the linear and robust models. Thus, the use of robust regression for the self-care and adaptive skills domains was based on the departure of the residuals from normality rather than on the presence of highly influential observations. For the comorbidity domain, the residuals of the linear model departed markedly from normality (both *p* < 0.001; maximum Cook’s distance, 0.019) and formed two parallel bands reflecting the binary scoring of this item; this domain was therefore analyzed using binary logistic regression.

The associations between the independent variables and the total RAKE-T score were examined using linear regression analysis, and the model was statistically significant (F = 4.954, *p* < 0.001). Each one-year increase in age was associated with a 0.208-point decrease in the total RAKE-T score (*p* = 0.033). Patients with a university education or higher had a 14.503-point higher total RAKE-T score than those with a high school education or lower (*p* < 0.001). The associations of the other variables with the total RAKE-T score were not statistically significant (*p* > 0.05).

The associations between the independent variables and the disease knowledge domain were examined using linear regression analysis, and the model was statistically significant (F = 4.806, *p* < 0.001). Each one-year increase in age was associated with a 0.045-point decrease in the disease knowledge score (*p* = 0.033). Patients with a university education or higher had a 2.841-point higher disease knowledge score than those with a high school education or lower (*p* < 0.001). In addition, with village residence as the reference category, patients living in a district had a 2.254-point higher disease knowledge score (*p* = 0.024). The associations of the other variables were not statistically significant (*p* > 0.05).

The associations between the independent variables and the pharmacological treatments domain were examined using linear regression analysis, and the model was statistically significant (F = 4.373, *p* < 0.001). Patients with a university education or higher had a 4.915-point higher pharmacological treatments score than those with a high school education or lower (*p* < 0.001). The associations of the other variables were not statistically significant (*p* > 0.05).

The associations between the independent variables and the non-pharmacological treatments domain were examined using linear regression analysis, and the model was statistically significant (F = 2.539, *p* = 0.009). Patients with a university education or higher had a 2.263-point higher non-pharmacological treatments score than those with a high school education or lower (*p* = 0.002). The associations of the other variables were not statistically significant (*p* > 0.05). Detailed results for the total RAKE-T score and the disease knowledge, pharmacological treatments, and non-pharmacological treatments domains are presented in Table 4.

The associations between the independent variables and the self-care domain were examined using robust regression analysis. Educational status was not significantly associated with the self-care score (β = 1.068, *p* = 0.054). With village residence as the reference category, patients living in a district had a 1.969-point higher self-care score (*p* = 0.011), and those living in a city had a 1.588-point higher self-care score (*p* = 0.018). The associations of the other variables were not statistically significant (*p* > 0.05).

The associations between the independent variables and the adaptive skills domain were examined using robust regression analysis. Patients with a university education or higher had a 3.372-point higher adaptive skills score than those with a high school education or lower (*p* < 0.001). In addition, with village residence as the reference category, patients living in a district had a 2.717-point higher adaptive skills score (*p* = 0.012), and those living in a city had a 1.898-point higher adaptive skills score (*p* = 0.041). The associations of the other variables were not statistically significant (*p* > 0.05). Detailed results for the self-care and adaptive skills domains are presented in Table 5.

The comorbidity item was answered correctly (score 2.22) by 111 (48.3%) patients and incorrectly or with “I don’t know” (score 0) by 119 (51.7%) patients. In the multivariable binary logistic regression analysis, none of the independent variables was significantly associated with a correct response to the comorbidity item (all *p* > 0.05; Cox & Snell R^2^ = 0.032; Nagelkerke R^2^ = 0.043). Detailed results are presented in Table 6.

In the sensitivity analysis additionally adjusting for recruitment center (reference: Kars), no statistically significant association with recruitment center was observed in any of the seven models (all *p* > 0.05). For the primary outcome, the association between age and the total RAKE-T score, which was statistically significant in the main analysis (β = −0.208, 95% CI −0.399 to −0.017, *p* = 0.033), was at the threshold of statistical significance after adjustment for center (β = −0.194, 95% CI −0.389 to 0.000, *p* = 0.050). After adjustment for center, compared with village residence, district residence remained associated with higher disease knowledge (β = 2.249, 95% CI 0.128 to 4.369, *p* = 0.038), self-care (β = 2.133, 95% CI 0.505 to 3.762, *p* = 0.011), and adaptive skills (β = 2.492, 95% CI 0.222 to 4.763, *p* = 0.032) scores, and city residence remained associated with higher self-care scores (β = 1.635, 95% CI 0.185 to 3.084, *p* = 0.027), whereas the association between city residence and adaptive skills scores was no longer statistically significant (β = 1.696, 95% CI −0.321 to 3.713, *p* = 0.099). In addition, university education, which was not significantly associated with self-care scores in the main analysis (β = 1.068, 95% CI −0.019 to 2.155, *p* = 0.054), was associated with higher self-care scores in the center-adjusted model (β = 1.118, 95% CI 0.022 to 2.214, *p* = 0.046). Because this association reached only borderline significance in a sensitivity analysis and the lower confidence limit was close to zero, it should be interpreted with caution; the interpretation of the present study is based on the main analysis presented in Table 4, Table 5 and Table 6.

## 4. Discussion

In this study, disease knowledge was assessed using RAKE-T in patients with RA receiving bDMARD/tsDMARD therapy, and sociodemographic and clinical factors potentially associated with disease knowledge were investigated. Older age was associated with a lower level of disease knowledge, whereas patients with a university education or higher had higher total RAKE-T scores as well as higher scores in the disease knowledge, pharmacological treatments, non-pharmacological treatments, and adaptive skills domains. In addition, compared with patients living in a village, those living in a district had higher disease knowledge, self-care, and adaptive skills scores, and those living in a city had higher self-care and adaptive skills scores. In contrast, disease duration, duration of biologic therapy, sex, marital status, and history of previous biologic agent use were not significantly associated with disease knowledge. These findings highlight the importance of assessing disease knowledge in routine clinical practice and considering the need for patient education, particularly among older patients, those with lower educational levels, and those living in rural areas.

Assessing disease knowledge in RA is important for planning patient education and supporting self-management. Indeed, EULAR emphasizes that patient education is an integral component of the standard of care for individuals with inflammatory arthritis [2]. Accordingly, various instruments have been developed to assess patients’ disease-related knowledge. Among these, RAKE, developed by Rodère et al. [3], is a reliable and comprehensive instrument that assesses essential knowledge regarding the disease, treatment, self-management, and treatment safety. Developed in accordance with contemporary treatment approaches and patient needs, RAKE has been proposed as a tool for identifying patients’ educational needs and evaluating the effectiveness of educational interventions. However, the number of studies using RAKE in different populations remains limited. These include studies evaluating the cultural adaptation and validation of the instrument, as well as studies assessing patients’ knowledge levels and the effects of educational interventions [4,5].

Among the sociodemographic factors examined, older age was associated with lower total RAKE-T scores as well as lower disease knowledge domain scores. Each one-year increase in age was associated with a 0.208-point lower total RAKE-T score (β = −0.208, 95% CI −0.399 to −0.017) and a 0.045-point lower disease knowledge score (β = −0.045, 95% CI −0.085 to −0.004). Although these confidence intervals did not include zero, the per-year differences were small and the upper limits of the intervals were close to zero; therefore, the magnitude of this association should be interpreted with caution. This finding is in line with the original RAKE validation study, in which younger age was associated with better knowledge [3], and with Abdulqasim et al., who reported that increasing age was associated with lower odds of good disease-related knowledge [6]. In contrast, Cortez et al. found no significant relationship between age and disease knowledge [8], suggesting that this association may not be uniformly observed across different populations and settings. The relationship between age and disease-related knowledge in RA may be multifactorial, potentially reflecting generational differences in health literacy, differences in how disease-related information is accessed or retained over time, or variation in engagement with newer educational resources across age groups. Regardless of the underlying mechanism, this finding suggests that older patients may represent a group with potentially greater educational needs and supports the value of considering age when planning and periodically reassessing individualized patient education during routine clinical follow-up.

Educational level emerged as the sociodemographic factor most consistently associated with disease knowledge in our cohort: patients with a university education or higher had higher total RAKE-T scores and higher scores in the disease knowledge, pharmacological treatments, and non-pharmacological treatments domains, and also scored higher in the adaptive skills domain than patients with a high school education or lower, whereas the difference in the self-care domain was not statistically significant. The magnitude of the association with education was most evident for the total RAKE-T score (β = 14.503, 95% CI 7.894 to 21.113); the entire confidence interval was above zero, but its width indicates uncertainty regarding the exact size of the difference. At the domain level, the estimates were also positive, for example, for the pharmacological treatments (β = 4.915, 95% CI 2.318 to 7.512) and adaptive skills (β = 3.372, 95% CI 1.852 to 4.893) domains. In contrast, the confidence interval for the self-care domain included zero (β = 1.068, 95% CI −0.019 to 2.155). Whether differences of these magnitudes are clinically meaningful cannot be determined from the present data. This pattern is consistent with the original RAKE validation study, in which higher educational attainment was associated with higher knowledge scores [3], and with the Turkish validation study of RAKE-T, in which patients with lower educational levels had significantly lower knowledge scores [4]. A similar positive association between education and disease knowledge has also been reported using other assessment instruments [6]. In contrast, some studies did not find a significant association between educational level and disease knowledge [7,8,9,11]; in one of these, the overall level of disease knowledge was low in the majority of the sample, which may have constrained the ability to detect differences by education [9], while in another, most participants had at least a secondary level of education, which may have narrowed the range over which an educational gradient could be observed [11]. Taken together, these observations may reflect the role of health literacy and general educational background in facilitating the comprehension and retention of disease-related information, including information provided verbally or in written form during routine care. Clinically, this finding underscores the potential value of tailoring the format, complexity, and delivery method of patient education to each patient’s educational background, rather than applying a uniform educational approach to all patients.

**Figure 1:**
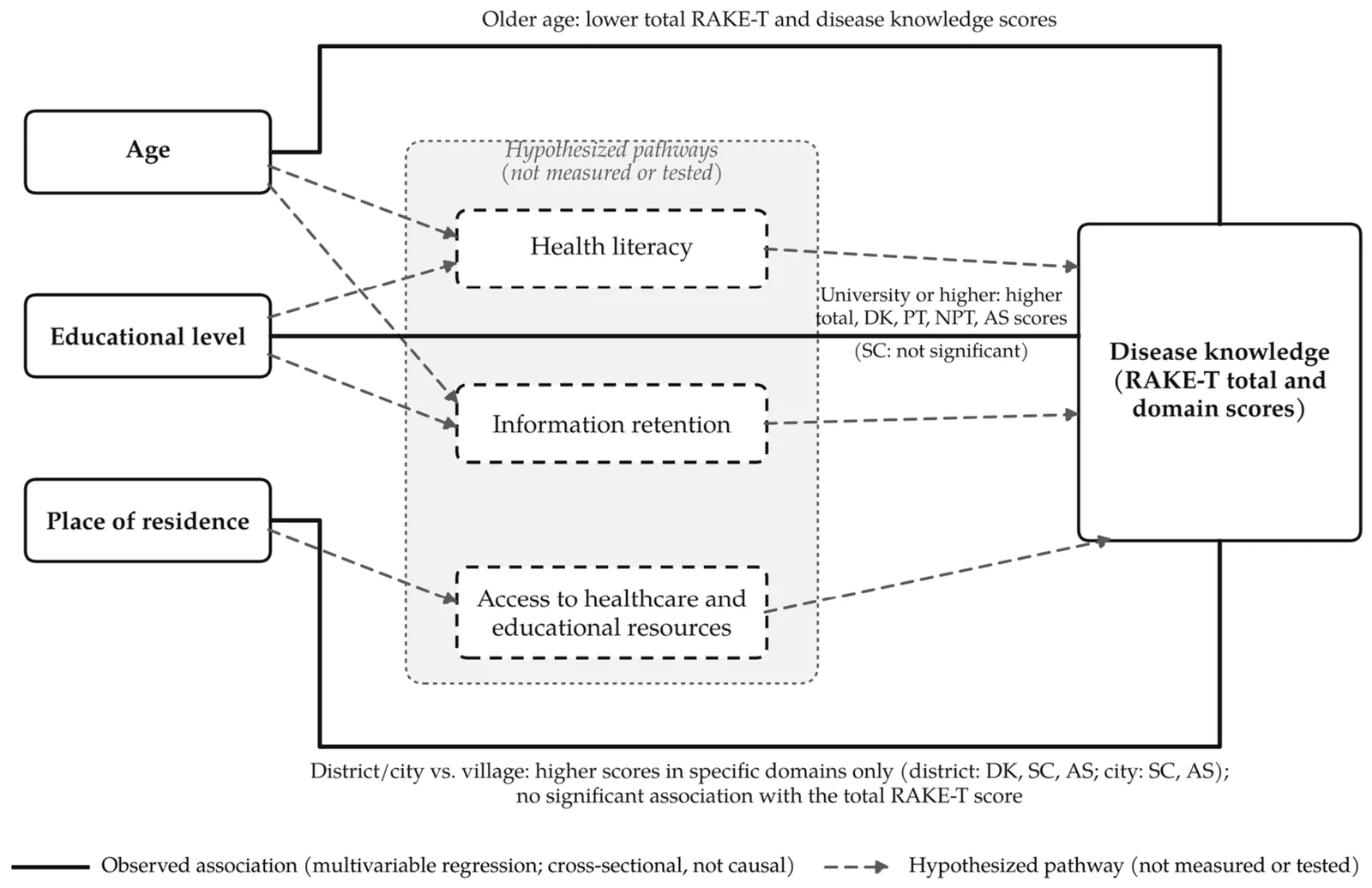
Conceptual diagram of the observed associations and hypothesized pathways linking age, educational level, and place of residence with disease knowledge. Solid lines indicate associations observed in the multivariable regression analyses of the present cross-sectional study (Table 4 and Table 5) and do not imply causality: older age was associated with lower total RAKE-T and disease knowledge scores; university education or higher was associated with higher total RAKE-T, disease knowledge, pharmacological treatments, non-pharmacological treatments, and adaptive skills scores, whereas the difference in self-care scores was not statistically significant; and, compared with village residence, living in a district was associated with higher disease knowledge, self-care, and adaptive skills scores, and living in a city was associated with higher self-care and adaptive skills scores, with no significant association between place of residence and the total RAKE-T score. Dashed arrows indicate hypothesized pathways through health literacy, information retention, and access to healthcare and educational resources; these factors were not measured, and these pathways were not tested in the present study. The shaded area and dashed-outline boxes represent hypothesized, unmeasured mechanisms, and bold text indicates the main study variables and the outcome. Abbreviations: DK, disease knowledge; PT, pharmacological treatments; NPT, non-pharmacological treatments; SC, self-care; AS, adaptive skills.

Place of residence was also related to disease knowledge in our cohort: compared with patients living in a village, those living in a district had higher disease knowledge, self-care, and adaptive skills domain scores, and those living in a city had higher self-care and adaptive skills domain scores. Compared with village residence, living in a district was associated with higher disease knowledge (β = 2.254, 95% CI 0.294 to 4.215), self-care (β = 1.969, 95% CI 0.457 to 3.481), and adaptive skills (β = 2.717, 95% CI 0.616 to 4.819) scores, and living in a city was associated with higher self-care (β = 1.588, 95% CI 0.271 to 2.906) and adaptive skills (β = 1.898, 95% CI 0.076 to 3.720) scores. However, these associations were limited to specific domains and were not observed for the total RAKE-T score. The wide confidence intervals, some with lower limits close to zero, and the small village reference group (*n* = 23) indicate that these estimates are imprecise; therefore, these findings should be considered exploratory. Literature directly examining place of residence as a determinant of RA-related disease knowledge is limited, which restricts direct comparison with previous studies. Differences according to place of residence may potentially reflect variation in access to specialized rheumatology care or educational resources; however, these factors were not directly measured in our study. Therefore, these possible explanations should be considered hypothesis-generating rather than established mechanisms. The observed associations and these hypothesized pathways, which were not measured or tested in the present study, are summarized in Figure 1.

Neither disease duration nor duration of biologic therapy was significantly associated with total RAKE-T scores or with any RAKE-T domain score in our cohort. This finding contrasts with the original RAKE validation study, in which longer disease duration was positively correlated with long-form RAKE scores [3], and with a subsample known-groups analysis in the Turkish validation study of RAKE-T, in which patients with a disease duration of five years or longer had higher scores than those with shorter disease duration [4]. However, our finding is consistent with previous studies using other knowledge assessment instruments, which also reported no significant association between disease duration and disease-related knowledge [6,7,8]. The lack of a statistically significant association with disease duration in our cohort should be interpreted cautiously and does not exclude the possibility of an association. All patients had received standardized education regarding treatment administration, adherence, adverse effects, and follow-up at the time biologic therapy was initiated; however, in the absence of structured reinforcement or periodic reassessment thereafter, the knowledge conveyed at treatment initiation may not necessarily be associated with higher scores among patients with a longer subsequent disease course. This observation carries a clinically meaningful implication: greater disease knowledge cannot be assumed on the basis of a longer disease duration or a longer duration of biologic therapy alone. Rather than being regarded as a one-time intervention delivered at treatment initiation, patient education may warrant periodic reassessment throughout follow-up, supporting the potential value of repeated and tailored educational strategies rather than reliance on treatment experience or time since diagnosis alone.

Sex, marital status, and history of previous biologic agent use were not significantly associated with total RAKE-T scores or with any domain score in our study. The lack of a significant association with sex contrasts with previous studies reporting higher disease knowledge among female patients [3,5,9], but is consistent with other studies in which sex was not significantly associated with disease knowledge [6,7]. Similarly, the absence of a significant association between marital status and disease knowledge is consistent with a previous study using RAKE-T, which also reported no significant relationship between marital status and disease knowledge [5]. Living arrangements, a related but distinct social characteristic, have also been examined in the literature; in the original RAKE validation study, disease knowledge did not differ significantly between patients living alone and those living with others [3]. However, living arrangements or whether patients lived alone were not specifically assessed in our study. We are not aware of previous studies specifically examining the relationship between history of previous biologic agent use and RA-related disease knowledge, which limits direct comparison for this variable. Overall, factors associated with disease knowledge in the present study, such as age, educational level, and place of residence, may warrant particular consideration when assessing patients’ educational needs.

Accordingly, periodic reassessment of educational needs during routine follow-up may be valuable, with particular consideration given to older patients, those with lower educational attainment, and patients who may have additional educational needs related to their place of residence. During routine rheumatology visits, clinicians could briefly reassess patients’ understanding of their disease and treatment, identify knowledge gaps, and provide targeted educational reinforcement, particularly for older patients and those with lower educational attainment. Tailoring the content and delivery of patient education to individual characteristics may therefore be more appropriate than applying a uniform educational approach to all patients.

This study has several strengths. Its multicenter design, involving four centers located in different geographic regions and sociodemographically diverse patient populations, may enhance the generalizability of our findings. In addition, the assessment of both total RAKE-T scores and individual domain scores allowed a more detailed evaluation of the factors associated with different aspects of disease knowledge. Another strength is the specific focus on patients receiving bDMARD/tsDMARD therapy, a population in whom the assessment of disease- and treatment-related knowledge is particularly relevant given the specific information required regarding treatment administration, safety, and monitoring. Finally, as RAKE is a relatively recently developed instrument and studies applying it across different populations remain limited, our study provides additional data to the existing literature in this field.

This study also has several limitations. Its cross-sectional design does not allow causal inference, and the associations identified should therefore be interpreted as observational rather than as evidence of cause-and-effect relationships. In addition, disease activity measures such as DAS28, CDAI, or SDAI were not assessed, precluding evaluation of the potential relationship between disease activity and disease knowledge in our cohort. Therefore, the possible influence of disease activity on disease knowledge cannot be determined from the present data, and we cannot exclude the possibility that unmeasured disease activity may have confounded some of the observed associations. Moreover, health literacy was not assessed using a validated instrument, and functional status was not evaluated. These factors, as well as other unmeasured potential confounders, may have influenced the observed associations; however, their possible effects could not be determined from the present data. The comorbidity domain consists of a single item and consequently has a restricted score distribution, with only two possible values (0 or 2.22); for this reason, it was analyzed as a binary outcome using logistic regression, although its limited variability still restricts the information available for this domain. Therefore, the nonsignificant findings for this domain should be interpreted with caution. Furthermore, although marital status was recorded, patients’ living arrangements and whether they lived alone were not specifically assessed; therefore, the potential relationships of these social characteristics with disease knowledge could not be examined. In addition, the relatively small number of patients residing in villages (*n* = 23) limits the precision of residence-related comparisons, and these domain-specific findings should be considered exploratory.

## 5. Conclusions

In conclusion, among patients with RA receiving bDMARD/tsDMARD therapy, disease knowledge assessed using RAKE-T was associated with specific sociodemographic characteristics, particularly older age and lower educational level, with additional domain-specific differences according to place of residence. In contrast, disease duration and duration of biologic therapy were not significantly associated with disease knowledge in the present study. These findings suggest that longer disease or biologic treatment experience should not be assumed to correspond to greater disease-related knowledge and highlight the importance of assessing patients’ educational needs during routine follow-up. Tailoring patient education to individual characteristics such as age, educational level, and place of residence may be beneficial. Further longitudinal studies incorporating measures of disease activity are needed to clarify how disease-related knowledge evolves over the course of RA treatment and to inform the content and timing of educational interventions in this population.

## Figures and Tables

**Table 1:** Demographic and Clinical Characteristics of the Study Population.

Characteristic	Mean ± SD	Median (Min–Max)
Age (years)	54.06 ± 13.46	57 (18–77)
Number of Children	2.04 ± 1.57	2 (0–9)
Disease Duration (months)	172.3 ± 105.46	168 (6–516)
Duration of Biologic Therapy (months)	55.7 ± 52.98	36 (3–252)
	*n*	%
Sex		
Female	177	77
Male	53	23
Educational Status		
Illiterate	16	7
Literate	21	9.1
Primary School	88	38.3
Middle School	30	13
High School	35	15.2
University or Higher	40	17.4
Educational Status		
High School or Lower	190	82.6
University or Higher	40	17.4
Marital Status		
Married	178	77.4
Single	52	22.6
Place of Residence		
Village	23	10
District	44	19.1
City	163	70.9
Children		
No	30	13
Yes	200	87
Smoking Status		
No	175	76.1
Yes	55	23.9
Family History of Rheumatic Disease		
No	130	56.5
Yes	100	43.5
Deformity		
No	157	68.3
Yes	73	31.7
Disease Duration		
<5 years	31	13.5
≥5 years	199	86.5
Previous Biologic Therapy		
No	128	55.7
<Yes	102	44.4

SD, standard deviation; Min, minimum; Max, maximum.

**Table 2:** bDMARD and tsDMARD Treatment Agents Used by the Study Population.

Treatment Agent	No	Yes
ADA	169 (73.5)	61 (26.5)
RTX	175 (76.1)	55 (23.9)
TCZ	198 (86.1)	32 (13.9)
ETN	207 (90)	23 (10)
GOL	215 (93.5)	15 (6.5)
CZP	221 (96.1)	9 (3.9)
TOFA	221 (96.1)	9 (3.9)
ABA	221 (96.1)	9 (3.9)
UPA	222 (96.5)	8 (3.5)
IFX	224 (97.4)	6 (2.6)
BARI	227 (98.7)	3 (1.3)

Abbreviations: ABA, abatacept; ADA, adalimumab; BARI, baricitinib; CZP, certolizumab pegol; ETN, etanercept; GOL, golimumab; IFX, infliximab; RTX, rituximab; TCZ, tocilizumab; TOFA, tofacitinib; UPA, upadacitinib. n, number of patients; %, percentage.

**Table 3:** Descriptive Statistics of the Total RAKE-T Score and Domain Scores.

Variable	Mean ± SD	Median (Min–Max)
Total RAKE-T Score	61.77 ± 19	62.22 (15.55–100)
Disease Knowledge	14.86 ± 4.06	15.55 (2.22–22.22)
Pharmacological Treatments	17.81 ± 7.39	17.77 (0–31.11)
Non-Pharmacological Treatments	8.81 ± 3.83	8.88 (0–15.56)
Comorbidity	1.07 ± 1.11	0 (0–2.22)
Self-Care	7.02 ± 3.12	6.66 (0–11.11)
Adaptive Skills	12.37 ± 4.54	13.33 (0–17.77)

Abbreviations: SD, standard deviation; Min, minimum; Max, maximum.

**Table 4:** Factors Associated with Total RAKE-T Score and Disease Knowledge, Pharmacological Treatments, and Non-Pharmacological Treatments Domain Scores.

Variable	Total	DK	PT	NPT
Intercept	60.444 (45.964–74.924) **<0.001**	14.878 (11.773–17.982) **<0.001**	16.531 (10.842–22.22) **<0.001**	10.088 (7.038–13.137) **<0.001**
Age	−0.208 (−0.399–−0.017) **0.033**	−0.045 (−0.085–−0.004) **0.033**	−0.07 (−0.145–0.005) 0.066	−0.032 (−0.072–0.008) 0.118
Disease Duration	0.013 (−0.011–0.038) 0.289	0 (−0.005–0.005) 0.958	0.008 (−0.002–0.018) 0.108	0.001 (−0.005–0.006) 0.821
Duration of Biologic Therapy	−0.001 (−0.049–0.047) 0.974	−0.003 (−0.014–0.007) 0.525	0 (−0.019–0.019) 0.970	0.006 (−0.004–0.016) 0.269
Sex	3.57 (−2.081–9.221) 0.214	1.063 (−0.148–2.275) 0.085	1.862 (−0.358–4.082) 0.100	0.201 (−0.989–1.392) 0.739
Educational Status	14.503 (7.894–21.113) **<0.001**	2.841 (1.424–4.258) **<0.001**	4.915 (2.318–7.512) **<0.001**	2.263 (0.871–3.655) **0.002**
Marital Status	−2.555 (−8.182–3.071) 0.372	−0.675 (−1.882–0.531) 0.271	−1.22 (−3.431–0.991) 0.278	−0.472 (−1.657–0.713) 0.433
Place of Residence				
District	8.224 (−0.921–17.368) 0.078	2.254 (0.294–4.215) **0.024**	1.454 (−2.139–5.047) 0.426	0.567 (−1.359–2.493) 0.562
City	6.273 (−1.66–14.207) 0.121	1.43 (−0.27–3.131) 0.099	2.336 (−0.781–5.453) 0.141	−0.35 (−2.021–1.32) 0.680
Previous Biologic Therapy	2.247 (−2.678–7.172) 0.370	0.709 (−0.347–1.764) 0.187	1.004 (−0.931–2.939) 0.308	−0.029 (−1.066–1.008) 0.957
R^2^	0.169	0.164	0.152	0.094
Adjusted R^2^	0.135	0.130	0.117	0.057
F	4.954	4.806	4.373	2.539
*p* (model)	**<0.001**	**<0.001**	**<0.001**	**0.009**

Values are presented as unstandardized β coefficients (95% confidence intervals) and *p* values. Abbreviations: DK, Disease Knowledge; PT, Pharmacological Treatments; NPT, Non-Pharmacological Treatments. Age is expressed in years; Disease Duration and Duration of Biologic Therapy are expressed in months. Reference categories: male for Sex, high school or lower for Educational Status, single for Marital Status, village for Place of Residence, and no for Previous Biologic Therapy. Model fit statistics (R^2^, adjusted R^2^, F, and model *p* value) were obtained from the linear regression models. Bold values indicate statistical significance (*p* < 0.05).

**Table 5:** Factors Associated with Self-Care and Adaptive Skills Domain Scores.

Variable	SC	AS
Intercept	6.459 (4.274–8.645) **<0.001**	11.36 (8.32–14.4) **<0.001**
Age	−0.024 (−0.056–0.007) 0.129	−0.024 (−0.068–0.02) 0.292
Disease Duration	0.001 (−0.003–0.005) 0.533	0.001 (−0.005–0.007) 0.684
Duration of Biologic Therapy	0.001 (−0.007–0.009) 0.730	0 (−0.011–0.012) 0.939
Sex	−0.117 (−1.051–0.817) 0.806	−0.532 (−1.834–0.769) 0.421
Educational Status	1.068 (−0.019–2.155) 0.054	3.372 (1.852–4.893) **<0.001**
Marital Status	0.004 (−0.929–0.937) 0.993	0.324 (−0.979–1.628) 0.624
Place of Residence		
District	1.969 (0.457–3.481) **0.011**	2.717 (0.616–4.819) **0.012**
City	1.588 (0.271–2.906) **0.018**	1.898 (0.076–3.72) **0.041**
Previous Biologic Therapy	0.095 (−0.72–0.91) 0.818	−0.219 (−1.352–0.913) 0.703
Weighted R^2^	0.080	0.147
Weighted adjusted R^2^	0.047	0.116

Values are presented as unstandardized β coefficients (95% confidence intervals) and *p* values. Abbreviations: SC, Self-Care; AS, Adaptive Skills. Age is expressed in years; Disease Duration and Duration of Biologic Therapy are expressed in months. Reference categories: male for Sex, high school or lower for Educational Status, single for Marital Status, village for Place of Residence, and no for Previous Biologic Therapy. Models were estimated using robust regression (Huber M-estimator; MASS::rlm). Because robust regression does not provide a standard R^2^, model fit was assessed by fixing the final Huber M-estimator observation weights and refitting the model with weighted least squares (WLS); the weighted R^2^ and weighted adjusted R^2^ obtained from this weighted model are presented for descriptive purposes only. No global F test or model *p* value is reported for the robust regression models because the WLS refit does not provide a valid overall significance test for the original robust regression. WLS, weighted least squares. Bold values indicate statistical significance (*p* < 0.05).

**Table 6:** Factors Associated with a Correct Response to the Comorbidity Item (Binary Logistic Regression).

Variable	Score 0 (*n* = 119)	Score 2.22 (*n* = 111)	OR (95% CI)	*p*
Age	54.02 ± 13.66	54.11 ± 13.32	1.003 (0.981–1.025)	0.776
Disease Duration	165.19 ± 106.75	179.91 ± 104.01	1.002 (0.999–1.005)	0.163
Duration of Biologic Therapy	57.26 ± 54.62	54.04 ± 51.35	0.996 (0.991–1.002)	0.196
Sex				
Female	93 (52.5)	84 (47.5)	0.873 (0.457–1.666)	0.681
Male	26 (49.1)	27 (50.9)	Reference	
Educational Status				
High School or Lower	101 (53.2)	89 (46.8)	Reference	
University or Higher	18 (45.0)	22 (55.0)	1.420 (0.665–3.033)	0.365
Marital Status				
Married	91 (51.1)	87 (48.9)	1.086 (0.570–2.071)	0.802
Single	28 (53.8)	24 (46.2)	Reference	
Place of Residence				
Village	13 (56.5)	10 (43.5)	Reference	
District	27 (61.4)	17 (38.6)	0.779 (0.271–2.238)	0.643
City	79 (48.5)	84 (51.5)	1.315 (0.531–3.255)	0.554
Previous Biologic Therapy				
No	71 (55.5)	57 (44.5)	Reference	
Yes	48 (47.1)	54 (52.9)	1.343 (0.765–2.356)	0.305

Values for Score 0 and Score 2.22 are presented as mean ± SD for continuous variables and as *n* (%) for categorical variables, with percentages calculated within each category of the independent variable. ORs (95% CIs) and *p* values were obtained from a multivariable binary logistic regression model including all listed variables; the OR represents the odds of a correct response to the comorbidity item (score 2.22) versus an incorrect or “I don’t know” response (score 0). Age is expressed in years; Disease Duration and Duration of Biologic Therapy are expressed in months. Model fit: Cox & Snell R^2^ = 0.032; Nagelkerke R^2^ = 0.043. Abbreviations: OR, odds ratio; CI, confidence interval; SD, standard deviation.

## Data Availability

The data that support the findings of this study are available from the corresponding author upon reasonable request. The data are not publicly available due to privacy and ethical considerations.

## Abbreviations

The following abbreviations are used in this manuscript:

ABA	Abatacept
ACR	American College of Rheumatology
ADA	Adalimumab
AS	Adaptive Skills
BARI	Baricitinib
bDMARD	Biologic Disease-Modifying Antirheumatic Drug
CDAI	Clinical Disease Activity Index
CZP	Certolizumab Pegol
DAS28	Disease Activity Score 28
DK	Disease Knowledge
ETN	Etanercept
EULAR	European Alliance of Associations for Rheumatology
GOL	Golimumab
IFX	Infliximab
Max	Maximum
Min	Minimum
NPT	Non-Pharmacological Treatments
OR	Odds Ratio
PT	Pharmacological Treatments
RA	Rheumatoid Arthritis
RAKE	Rheumatoid Arthritis Knowledge Assessment Questionnaire
RAKE-T	Turkish Version of the Rheumatoid Arthritis Knowledge Assessment Questionnaire
RTX	Rituximab
SC	Self-Care
SD	Standard Deviation
SDAI	Simplified Disease Activity Index
TCZ	Tocilizumab
TOFA	Tofacitinib
tsDMARD	Targeted Synthetic Disease-Modifying Antirheumatic Drug
UPA	Upadacitinib
